# Large-scale correlation analysis of deep venous thrombosis and gut microbiota

**DOI:** 10.3389/fcvm.2022.1025918

**Published:** 2022-11-07

**Authors:** Mingyi Yang, Pan Luo, Feng Zhang, Ke Xu, Ruoyang Feng, Peng Xu

**Affiliations:** ^1^Department of Joint Surgery, Honghui Hospital, Xi’an Jiaotong University, Xi’an, Shaanxi, China; ^2^Key Laboratory of Trace Elements and Endemic Diseases, National Health and Family Planning Commission, School of Public Health, Health Science Center, Xi’an Jiaotong University, Xi’an, Shaanxi, China

**Keywords:** thrombosis, deep venous, microbiota, genetic, prevention

## Abstract

**Objective:**

Although previous studies have shown that gut microbiota may be involved in the occurrence of deep venous thrombosis (DVT), the specific link between the two remains unclear. The present study aimed to explore this question from a genetic perspective.

**Materials and methods:**

Genome-wide association study (GWAS) summary data of DVT were obtained from the UK Biobank (*N* = 9,059). GWAS summary data of the gut microbiota were obtained from the Flemish Gut Flora Project (*N* = 2,223) and two German cohorts (FoCus, *N* = 950; PopGen, *N* = 717). All the participants were of European ancestry. Linkage disequilibrium score (LDSC) regression has great potential for analyzing the heritability of disease or character traits. LDSC regression was used to analyze the genetic correlation between DVT and the gut microbiota based on the GWAS summary data obtained from previous studies. Mendelian randomization (MR) was used to analyze the genetic causal relationship between DVT and the gut microbiota. We used the random effects inverse variance weighted, MR Egger, weighted median, simple mode, and weighted mode to perform MR analysis. We performed a sensitivity analysis of the MR analysis results by examining heterogeneity and horizontal pleiotropy.

**Results:**

Linkage disequilibrium score analysis showed that Streptococcaceae (correlation coefficient = −0.542, SE = 0.237, *P* = 0.022), Dialister (correlation coefficient = −0.623, SE = 0.316, *P* = 0.049), Streptococcus (correlation coefficient = −0.576, SE = 0.264, *P* = 0.029), and Lactobacillales (correlation coefficient = −0.484, SE = 0.237, *P* = 0.042) had suggestive genetic correlation with DVT. In addition, the MR analysis showed that Streptococcaceae had a positive genetic causal relationship with DVT (*P* = 0.027, OR = 1.005). There was no heterogeneity or horizontal pleiotropy in the MR analysis (*P* > 0.05).

**Conclusion:**

In this study, four gut microbes (Streptococcaceae, Dialister Streptococcus, Lactobacillales) had suggestive genetic correlations with DVT, and Streptococcaceae had a positive causal relationship with DVT. Our findings provide a new research direction for the further study of and prevention of DVT.

## Introduction

Deep venous thrombosis (DVT) is a venous thromboembolic (VTE) disease that includes superficial thrombophlebitis and pulmonary embolism (PE). DVT most often occurs in the deep veins of the lower extremities, but it can also occur in the deep visceral veins of the upper extremities and vena cava (1). DVT is a common complication in almost all inpatients and an important cause of PE and even death, with a higher incidence in patients with traumatic conditions (2, 3). DVT can occur due to internal diseases and as a complication of surgical procedures and is particularly common in patients undergoing orthopedic surgery (4–6). DVT is a common post-operative complication in patients undergoing major orthopedic surgery such as total hip replacement, total knee replacement, or hip fracture surgery (HFS) (5). In retrospective studies, the incidence of DVT after arthroscopic knee surgery was 0.24%, while in prospective studies, it was 2.9% (7). The incidence of DVT in patients with hemophilia undergoing major orthopedic surgery was reported to be 10% (5). The incidence of DVT after posterior spinal surgery has increased more rapidly than expected (8). DVT is a major hidden danger for medical diseases and perioperative management, which seriously threatens the life and health of patients. However, currently, there is no clear and effective treatment for DVT. The prevention of DVT is the main focus of clinicians, and a comprehensive and in-depth understanding of the related risk factors is key to preventing DVT.

Venous thrombosis (DVT, PE) is a common and serious disorder associated with both genetic and acquired risk factors. Genetic risk factors can be subdivided into strong, moderate, and weak. Strong risk factors include deficiencies in antithrombin, protein C, and protein S. Moderately strong are Factor V Leiden, prothrombin 20210A, non-O blood group, and fibrinogen 10034T. There are many weak genetic risk factors, including fibrinogen, factor XIII, and factor XI variants (9). DVT is a common complication in patients undergoing orthopedic surgery, and genetic risk factors and high heritability greatly increase the risk of DVT (10). Inherited hypercoagulable states are present in the majority of patients with VTE diseases (11). DVT most often arises from the convergence of multiple genetic and acquired risk factors, with an estimated incidence of 56–160 cases per 100,000 population per year (12). Studies have found that women with hereditary antithrombin deficiency have a significantly increased risk of venous thromboembolism after pregnancy and a significantly increased risk of DVT during their second pregnancy (13, 14). Methylenetetrahydrofolate reductase (MTHFR) gene mutations may cause an imbalance in vasorelaxation and vasoconstriction factors, leading to the occurrence of DVT. The frequency of the MTHFR 677TT genotype may be related to DVT pathogenesis (15). It can be seen that genetic factors play an important role in the occurrence of DVT. Understanding the genetic traits related to DVT has potential value for further studies.

Gut microbiota is a bacterial community that colonizes the human gut and is interdependent with the human body throughout life. In recent years, the gut microbiota has been found to be related to immune, metabolic, and neurological characteristics; drug metabolism; and cancer (16). Environmental factors, such as diet and drug use, play important roles in the composition of the gut microbiota. Studies based on twins in families and within populations show that genetic components also play an important role in determining the composition of the gut microbiota and the proportion of bacteria in the gut (17). The gut microbiota is associated with many diseases, such as spondyloarthropathy (SpA), osteoarthritis (OA), rheumatoid arthritis (RA), and osteoporosis (OP) (17–20).

Deep venous thrombosis and PE are common causes of morbidity and mortality in VTE (21). Studies have found that several diseases with an increased risk of VTE, including DVT, are related to an imbalance in the gut microbiota characterized by a decrease in symbiotic anaerobic bacteria and an increase in the number of pathogenic bacteria, the most common of which is gram-negative Enterobacteriaceae (ENTERO) (22). Bacterial lipopolysaccharides (LPS) derived from the gut microbiota play an important role in hypercoagulability and venous thromboembolism. As one of the links between microbiota and hypercoagulability, LPS activates endothelial cells and platelets by binding toll-like receptors, leading to activation of the coagulation cascade (22). Microbiota metabolites, including trimethylamine N-oxide (TMAO), remain associated with post-incident VTE patients, highlighting the possible involvement of gut microbiota in VTE risk and relapse (21). Gut microbiota-dependent TMAO shows a U-shaped association with VTE (23). The composition of gut microbiota may affect the human coagulation system (24). Key determinants of thrombotic risk, FVIII and VWF levels, have also been shown to be controlled by gut microbiota-derived pathogen-associated molecular patterns (PAMPs) that can emanate from the gut space (25). Interaction of these gut-derived PAMPs with toll-like receptor 2 (TLR2) on hepatic sinusoidal endothelial cells induces increased VWF/FVIII secretion in mice. This effect was dependent on TLR2 and gut commensals, and VWF levels were significantly reduced in both TLR2-deficient and germ-free mice (26). A study found that several forms of vitamin K (menaquinones) are synthesized by Bacteroides, Enterobacter, Veillonella, and Eubacterium lentum, which are common members of the microbiota (intestinal microflora). Excessive vitamin K synthesized by the microbiota can be absorbed more than usual along the intestinal lymph vessels together with lipids, thus affecting the coagulation cascade and venous thrombosis (27). Although previous studies have shown a link between gut microbiota, thrombosis, and DVT, no studies have explored the genetic association of gut microbiota with DVT.

Linkage disequilibrium score (LDSC) regression has been applied to existing genome-wide association study (GWAS) summary data to assess the heritability of diseases and traits, which can correct for polygenic inheritance effects and confounding factors in GWASs. Mendelian randomization (MR) is a new strategy for investigating causation between different traits based on Mendel’s laws of inheritance (28). In MR studies, exposure is regarded as an intermediate phenotype, which is determined by genotype, and the differences in genotype [generally single-nucleotide polymorphisms (SNPs)] are used as instrumental variables (IVs) to study the association effect between genotypes and diseases to simulate the association between exposure and disease. Therefore, MR studies are not affected by the confounders of traditional epidemiological methods (such as retrospective studies) and reverse causality (29). In this study, we used LDSC and MR to analyze the genetic correlation between DVT and gut microbiota to broaden the research horizon on DVT and provide new research directions for DVT prevention.

## Materials and methods

### Genome-wide association study summary data of deep venous thrombosis

The GWAS summary data for DVT were obtained from the UK Biobank (UK Biobank fields: 20002). In short, the UK Biobank participants were of European ancestry. The UK Biobank contains the ancestry genetic association maps for 118 non-binary traits and 660 binary traits for a total of 452,264 participants (males and females). In total, 9,059 DVT cases and 443,205 controls were included in our study. The Affymetrix UK BiLEVE Axiom and Affymetrix UK Biobank Axiom arrays were used for genotyping. We excluded individuals who were identified by the UK Biobank as outliers based on either the genotyping missingness rate or heterogeneity, whose sex inferred from the genotypes did not match their self-reported sex, and who were not of European ancestry. Finally, we removed individuals with missingness >5% across variants that passed our quality control procedure and those that had a missing phenotype for 40 or more traits. An estimated 90 million genetic variants were identified in the Haplotype Reference Consortium, 1000 Genomes, and UK10K projects. After quality control, 62,394 genotype variants and 9,113,133 variable estimates were obtained. In previous studies, all participants signed an informed consent form and obtained approval from the ethics committee. Detailed information on genotyping, estimation and quality control can be found in a previous study (30).

### Genome-wide association study summary data of the gut microbiota

Genome-wide association study summary data of the gut microbiota were obtained from a previous study (31). In short, participants of European ancestry included those from the Flemish Gut Flora Project (FGFP) (*N* = 2,223) and two German cohorts (FoCus, *N* = 950; PopGen, *N* = 717). Previous studies have provided additional information on the age, sex, height, body mass index, and waist-to-hip ratio distribution of the FGFP cohort (31). SHAPEIT3 was used for genotyping the FGFP data. The UK10K and 1000 Genome Project phase 3 samples were used as reference panels, and IMPUTE4 was used for interpolation (32). According to the quality control of the genotype and microbiome data and considering the missing data for the covariates, a total of 2,223 individuals were identified, and a total of 7,711,310 SNPs were included after filtering. All single-nucleotide variants at an inclusive association score test threshold of *P* < 1 × 10^–5^ in the FGFP dataset were used in a targeted meta-analysis, including two independent German cohorts. An Illumina Omni Express + Exome array and Affymetrix Genome-Wide Human SNP Array 6.0 were used for genotyping the FoCus and PopGen cohorts, respectively. Previous studies have provided basic information on the age and sex of participants in the FoCus and PopGen cohorts (33, 34). Quality control and interpolation were performed for genotyping of the two cohorts. In previous studies, all participants signed an informed consent form and approval was obtained from the relevant ethics committee. Ultimately, 74 gut microbiota samples were included in our study. Detailed information regarding the data, genotyping, quality control, and imputation can be found in a previously published study (31).

### Linkage disequilibrium score regression

Linkage disequilibrium score estimates genome-wide genetic correlations only from GWAS summary data and is not affected by sample overlap (32). LDSC is essentially a linear regression, and its input data are the results of GWASs; the independent variable in the regression is the LD score of SNP sites, and the dependent variables are the core of the algorithm. A custom statistic conforming to the chi-square distribution was defined, and the relationship between the LD score and chi-square statistic was fitted using linear regression to determine whether there were confounding factors in the GWAS results. LDSC can calculate each variant’s ability to tag other variants locally, which means higher LD scores suggest a higher possibility of tag casual sites. When LDSC was used for genetic correlation estimation, it quantified the genetic covariance between two traits by regressing the product of the z-scores from two studies of traits against the LD score for each SNP (35). Both polygenicity and confounding biases, such as cryptic relatedness and population stratification, can yield an inflated distribution of test statistics in GWAS. However, the current methods cannot distinguish between inflation from a true polygenic signal and bias. The LDSC quantifies the contribution of each by examining the relationship between the test statistics and LD. The LDSC regression intercept can be used to estimate a more powerful and accurate correction factor than the genomic control (36).

The basic principle of the LDSC approach is to directly estimate heritability and genetic correlation from GWAS summary data using the deviation of the observed χ*^2^* test statistic for an SNP from its expected value under the null hypothesis of no association. An SNP tagging more of its neighbors, and thus having a higher LD score, is more likely to tag one or more causal sites that affect the phenotype. If genetic correlations are statistically and quantitatively significant, then we can determine that total phenotypic correlations cannot be fully attributed to environmental confounders (37). Under a polygenic model, in which effect sizes for variants are drawn independently from distributions with variance proportional to 1/(*p*(1 – *p*), where *p* is the minor allele frequency, the expected χ^2^ statistic of variant *j* is:


$$E\,\left[x^{2}|\ell_{j}\right]=N\,h^{2}\,\ell_{j}/M+N\,a+1$$

where *N* is the sample size, *M* is the number of SNPs, such that *h*^2^/*M* is the average heritability explained per SNP, *a* measure of the contribution of confounding biases, such as cryptic relatedness and population stratification, and


$$\ell_{j}=\Sigma_{k^{r^{2}}\,j\,k}$$

is the LD score of variant *j*, which measures the amount of genetic variation tagged with *j* (36). This relationship holds for meta-analyses and ascertained studies of binary phenotypes, in which case *h*^2^ is on the observed scale. Consequently, if we regress the χ^2^ statistics from GWAS against the LD score, the intercept minus one is an estimator of the mean contribution of the confounding bias to inflation in the test statistics (36).

In contrast to the genomic restricted maximum likelihood approach, the LDSC method does not require individual-level genotype data but instead uses GWAS summary statistics, regressing association test statistics of SNPs on their LD scores (38). The LD score of an SNP is the sum of LD *r*^2^ measured with all other SNPs and can be calculated in a reference sample of the same ethnicity when individual genotype data are not available for the GWAS sample under the assumption that the GWAS sample has been drawn from the same ethnic population as the reference sample used to calculate the LD scores (38). This method exploits the relationship between the association test statistic and the LD score expected under polygenicity. LDSC is a powerful tool for evaluating genetic correlations of complex diseases and traits using GWAS summary data.

According to the standard method recommended by the developer, LDSC analysis software^1^ was used to evaluate the genetic correlation between DVT and gut microbiota. A *P*-value of < 0.05 was considered suggestive of genetic correlations.

### Mendelian randomization analysis

We further performed an MR analysis on gut microbes that were found to be genetically related to DVT. Three assumptions of MR must be met to obtain impartial results: (1) the genetic IVs should have a strong link to the exposure; (2) the genetic IVs are not associated with confounders linked to the chosen exposure and outcome; and (3) genetic IVs influence the outcome only through exposure and not *via* other biological pathways (39).

To ensure the accuracy and robustness of the conclusions, we employed a series of quality control steps to select the valid IVs. First, we obtained SNPs associated with gut microbes (*P* < 1 × 10^–5^) (40). Second, because strong LD among the selected SNPs may lead to biased results, the clumping process (*r*^2^ < 0.001, clumping distance = 10,000 kb) was carried out to eliminate the LD between the included IVs (18). Third, we excluded SNPs associated with the outcome (DVT) (*P* < 1 × 10^––5^). Fourth, the PhenoScanner database^2^ was used to assess whether the selected SNPs were associated with confounders (41). We considered the risk factors and potential confounders of DVT (obesity, age, sex, smoking, physical activity, use of lipid-lowering therapy, Factor V Leiden, cancer, recent major surgery and study, and for women only the use of oral contraceptives, use of hormone-replacement therapy, and menopausal status) (42). Fifth, palindromic SNPs with intermediate allele frequency were excluded. Sixth, when SNPs were not available in the GWAS results, proxy SNPs were identified using the LDlink online platform.^3^

We used the random effects inverse variance weighted (IVW), MR Egger, weighted median, simple mode, and weighted mode to perform the MR analysis. Random effects IVW was the main method used, and the weighted median, simple mode, and weighted mode were used as supplementary methods. The MR analysis results were dominated by the random effects IVW. We used Cochran’s Q statistic for MR-IVW analyses and Rucker’s Q statistic for MR Egger analyses to detect heterogeneity, and *P*-value > 0.05 was considered to indicate no heterogeneity (43). We used the MR Egger method to assess the extent to which directional pleiotropy may affect risk estimates by intercept tests, and *P*-value > 0.05 was considered to indicate no horizontal pleiotropy (41). Because MR Egger may show lower accuracy in some cases, the MR pleiotropy residual sum and outlier (MR-PRESSO) method was also used to assess outlier SNPs and potential horizontal pleiotropy (41). The distortion test embedded in the MR-PRESSO analysis can detect outliers present in the MR analysis. The global test embedded in the MR-PRESSO analysis can detect horizontal pleiotropy, with *P*-value > 0.05 considered to indicate no horizontal pleiotropy (28).

## Results

### Genetic correlation estimation

**FIGURE 1:**
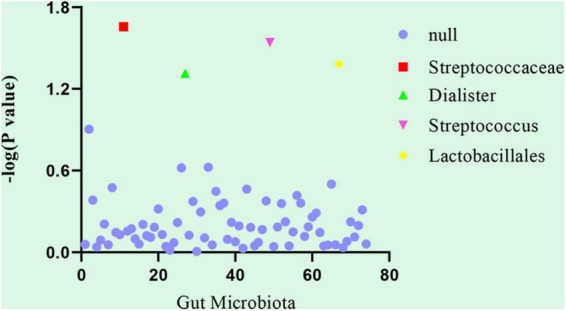
Genetic correlation analysis between deep venous thrombosis and gut microbiota. Abscissa: the number of gut microbiota, arranged in number order for the gut microbiota (74 gut microbiota). Ordinate: *P*-value of the results of the genetic correlation analysis between deep venous thrombosis and gut microbiota. Null: The linkage disequilibrium score regression showed the gut microbiota that had no suggestive genetic correlation with deep venous thrombosis, that is, ineffective gut microbiota (*P*-value > 0.05).

Linkage disequilibrium score was used to analyze the genetic correlation between DVT and gut microbiota. After LDSC regression analysis, we obtained the results of the genetic correlation evaluation between the 74 gut microbiota and DVT. The LDSC analysis results of the 74 gut microbiota samples and DVT are shown in Supplementary Table 1. Considering the multiple testing burden, we used a correlation threshold of 0.05/74 = 0.00068. However, in our LDSC analysis, no gut microbiota met this condition. In addition, our false discovery rate was not ideal, but it is a very strict statistical criterion. Therefore, we considered the gut microbiota with a *P*-value in the range of 0.00068–0.05 as a suggestive association. Among the 74 gut microbiota, four taxa had suggestive genetic correlations with DVT: Streptococcaceae (correlation coefficient = −0.542, SE = 0.237, *P* = 0.022), Dialister (correlation coefficient = −0.623, SE = 0.316, *P* = 0.049), Streptococcus (correlation coefficient = −0.576, SE = 0.264, *P* = 0.029), and Lactobacillales (correlation coefficient = −0.484, SE = 0.237, *P* = 0.042) (Table 1 and Figure 1).

**TABLE 1:** Genetic correlation between gut microbiota and deep venous thrombosis.

Gut microbiota		Correlation coefficient	SE	*P*-value	FDR
Streptococcaceae	Deep venous thrombosis	−0.542	0.237	0.022	0.899
Dialister		−0.623	0.316	0.049	0.899
Streptococcus		−0.576	0.264	0.029	0.899
Lactobacillales		−0.484	0.237	0.042	0.899

### Genetic causal estimation

After a series of quality controls and removing a smoking-related SNP (rs2952251), we finally obtained 16 SNPs as IVs for the MR analysis of Streptococcaceae and DVT, including three palindromic SNPs (rs395407, rs6563952, and rs76717940). We obtained 12 SNPs as IVs for MR analysis of Dialister and DVT, including one palindromic SNP (rs517089). We obtained 17 SNPs as IVs for the MR analysis of Streptococcus and DVT, including two palindromic SNPs (rs395407 and rs6563952). After removing a smoking-related SNP (rs2952251), we obtained 18 SNPs as IVs for the MR analysis of Lactobacillales and DVT, including four palindromic SNPs (rs111552159, rs1962325, rs74352383, and rs76717940). None of the SNPs were proxied.

**FIGURE 2:**
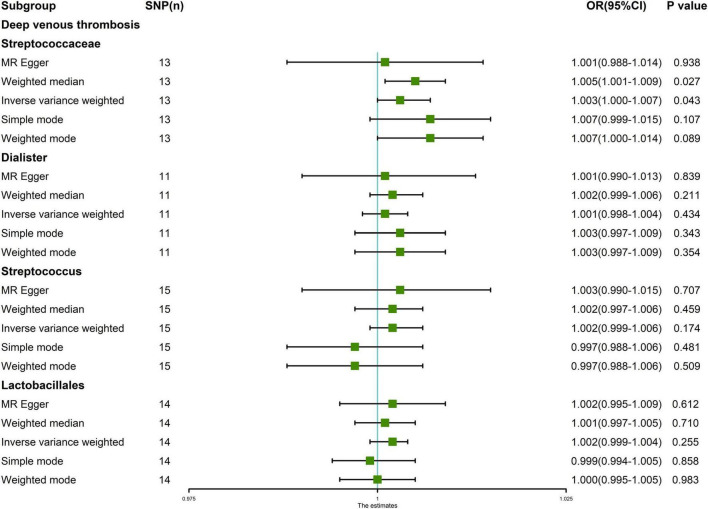
Mendelian randomization (MR) analysis results of the exposures (Streptococcaceae, Dialister, Streptococcus, and Lactobacillales) and outcomes (deep venous thrombosis). Five methods: random-effects inverse variance weighted, MR egger, weighted median, simple mode, and weighted mode.

**FIGURE 3:**
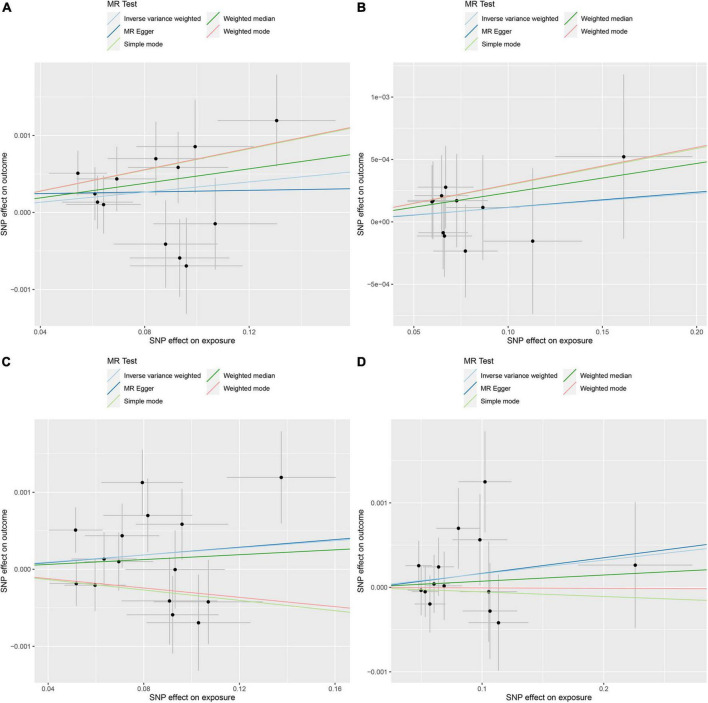
Mendelian randomization (MR) analysis of the exposures (Streptococcaceae, Dialister, Streptococcus, and Lactobacillales) and outcomes (deep venous thrombosis). **(A)** Scatter plot of Streptococcaceae and deep venous thrombosis; **(B)** scatter plot of Dialister and deep venous thrombosis; **(C)** scatter plot of Streptococcus and deep venous thrombosis; **(D)** scatter plot of Lactobacillales and deep venous thrombosis.

The random effects IVW results showed that Streptococcaceae (*P* = 0.043, OR = 1.003) had a positive genetic causal relationship with DVT. The weighted median results also showed that Streptococcaceae (*P* = 0.027, OR = 1.005) had a positive genetic causal relationship with DVT. The MR Egger, simple mode, and weighted mode analyses showed that Streptococcaceae had no genetic causal relationship with DVT (*P* > 0.05). In addition, random effects IVW, MR Egger, weighted median, simple mode, and weighted mode showed that Dialister, Streptococcus, and Lactobacillales had no genetic causal relationship with DVT (*P* > 0.05) (Figures 2, 3).

The MR-IVW and MR Egger tests showed that our MR analysis results had no heterogeneity (*P* > 0.05). The results of the MR Egger test of horizontal pleiotropy showed that our MR analysis results had no horizontal pleiotropy (*P* > 0.05). The MR-PRESSO global test results also showed that our MR analysis results showed no horizontal pleiotropy (*P* > 0.05). The MR-PRESSO distortion test results appeared to have no outliers (Table 2).

**TABLE 2:** Sensitivity analysis of the MR analysis results of gut microbiota and deep venous thrombosis.

		Heterogeneity test		Pleiotropy test	MR-PRESSO	
		Cochran’s *Q*-Test (*P*-value)	Rucker’s *Q*-Test (*P*-value)	Egger Intercept (*P*-value)	Distortion test	Global test
				
Exposure	Outcome	IVW	MR-Egger	MR-Egger	Outliers	*P-value*
Streptococcaceae	Deep venous thrombosis	0.356	0.296	0.673	NA	0.488
Dialister		0.987	0.975	0.989	NA	0.978
Streptococcus		0.107	0.077	0.978	NA	0.173
Lactobacillales		0.781	0.713	0.943	NA	0.919

## Discussion

Deep venous thrombosis is a common complication of various diseases and surgical procedures; in particular, DVT is a common complication of orthopedic surgery. There is currently no clear and effective preventive measure for DVT. The gut microbiota is a complex and dynamic ecological microbial community that settles in the human gut and is called the “forgotten organ” (44). For the first time, we studied the genetic correlation between DVT and gut microbiota based on GWAS summary data from a large population and found that Streptococcaceae, Dialister, Streptococcus, and Lactobacillales had a suggestive genetic correlation with DVT. There was a positive genetic causal relationship between Streptococcaceae and DVT. In this study, the genetic association of Streptococcaceae, Dialister, Streptococcus, and Lactobacillales with DVT may be related to the mechanism of DVT formation, and Streptococcaceae may be a high-risk factor for DVT. These findings contribute to the further study of DVT, and the regulation of Streptococcaceae may play a role in the prevention of DVT.

There are trillions of microbes in the human body that live in countless ecological environments in the host body and have the highest density in the gastrointestinal tract (45). The human gut is the natural habitat of a large and dynamic bacterial community, which is inseparable from human health. Gut microbiota is an important part of human life and plays an important role in the structure and function of the human body. Additionally, the gut microbiota plays an important role in carbohydrate metabolism, energy production, and the synthesis of cellular components. It can also process nutrients, promote the development of the immune system, and stimulate a variety of host activities (46). Individuals differ in their gut microbiota; however, in general, family members tend to have similar gut microbiota characteristics, which may be affected by genetic and environmental factors (e.g., diet) (47). The gut microbiota is the largest group of bacteria throughout the human body and undergoes a series of changes in the process of human growth and aging. Babies have a unique and unstable gut microbiota, and their dominant anaerobic bacteria include Bifidobacterium, Bacteroides, Clostridia, and Parabacteroides (48). With increasing age, the diversity of the gut microbiota increases, and the phylogenetic composition and function of this community tend to be stable. The bacterial composition of the adult intestinal epithelial barrier mainly includes Bacteroidetes, Firmicutes, Actinobacteria and Proteobacteria (49).

Thrombosis refers to the coagulation of blood or agglutination of some tangible components in blood to form solid masses, which are mainly caused by damage to vascular endothelial cells, abnormal blood flow, and increased blood coagulation. Although thrombus formation can have a hemostatic effect on ruptured blood vessels, in most cases, it can cause a series of adverse events in the body, such as blockage of blood vessels, embolism, heart valve deformation, and extensive bleeding. Thrombosis is caused by complex interactions between the coagulation system, the innate immune system, and inflammation. Inflammation is related to ecological disorders, increased intestinal permeability, and production of specific metabolites (22). Thrombosis is based on the cumulative effect of genetic and environmental risk factors, and its pathogenesis is complex (50). The gut microbiota is disturbed by various environmental and genetic factors, which can activate vascular endothelial cells, platelets, and inflammatory pathways of innate immune cells, thereby releasing various coagulation proteins and leading to a pre-thrombotic state (25). The intestinal epithelial barrier restricts the microbiota in the intestinal lumen. When inflammation, nutrition, antibiotics, and other factors impair the function of the lumen, the intestinal epithelial barrier allows intestinal microbial products and metabolites to enter the portal vein and then enter the systemic circulation. This leads to various pathological conditions, including potential thrombosis (51). There is a potential correlation between the gut microbiota and thrombosis, which also verifies the reliability of the results of this study.

One of our important findings in this study is that there is not only a suggestive genetic correlation but also a positive causal genetic relationship between Streptococcus and DVT. Streptococcaceae is an important group that includes three genera: Streptococcus, Lactococcus, and Lactovum. According to the data from “The Catalog of Life in 2013,” Streptococcus contains 67 species, Lactococcus contains five species, and Lactovum contains one species (52). Group A Streptococcus can not only trigger immune-mediated platelet activation through the M1 protein, leading to platelet aggregation and platelet-rich thrombosis, but also cause knee septic arthritis to progress to venous thrombosis in the tibia and fibula (53, 54). Streptococcus spp. may be correlated with thrombosis. Increasing evidence indicates that the gut microbiota is related to the pathogenesis of liver cirrhosis (LC) complications. The abundance of Streptococcaceae in the stool of patients with LC and patients with advanced LC complicated by hepatic encephalopathy is higher than that of healthy people, and Streptococcaceae is generally highly abundant at the family level (55, 56). The abundance of Streptococcaceae in the stool of patients with acute-on-chronic liver failure is also higher than that in healthy people (57). Portal vein thrombosis (PVT) is a well-known complication of LC caused by the stagnation of portal vein blood flow due to abdominal infection, surgery, trauma, hereditary or acquired pre-thrombosis disease state, and vascular endothelial injury (58). Compared with that in LC patients without PVT, the incidence of some prothrombin genotypes, including the Factor V Leiden G1691A mutation, methylenetetrahydrofolate reductase (MTHFR) C677T mutation, and the prothrombin G20210A mutation, is higher in LC patients with PVT (59). Studies have found that anticardiolipin antibodies are also common in LC patients with PVT and may be a potential risk factor for PVT caused by Bacteroides fragilis bacteremia (59). PVT in LC patients is related to genetics, thrombosis, and the gut microbiota. We can also infer that PVT caused by thrombosis in LC patients is potentially related to the gut microbiota and the abundance of Streptococcaceae in the gut. Previous studies have shown that Streptococcaceae are a high-risk factor for DVT. The results of this study indicate that Streptococcaceae had a positive genetic causal relationship with DVT, indicating that Streptococcaceae could promote the occurrence of DVT, which is consistent with the previous literature. The positive causal genetic relationship between Streptococcaceae and DVT provides a new research direction for the prevention of DVT.

Another important finding of this study is the suggestive genetic correlation between Dialister and DVT. Dialister is a non-motile, non-spore-forming, sugar-free obligate anaerobic, gram-negative coccus. There are currently four species of the genus Dialister: Dialister pneumosintes, Dialister invisus, Dialister micraerophilus, and Dialister propinicifaciens (60). Dialister produces propionic acid in the gut (61). Recent studies have shown that the abundance of Dialister was significantly reduced in pediatric patients with juvenile idiopathic arthritis, adult idiopathic nephrotic syndrome, spinal cord injury, autism spectrum disorder, depression, and Henoch-Schönlein purpura but was positively correlated with quality of life (62–67). Dialister pneumosintes is a small non-fermenting gram-negative anaerobic bacterium commonly found in the oral, nasopharynx, intestinal, and vaginal flora (68). It has been reported that three groups of blood cultures were performed on patients with postpartum vaginosis and ovarian venous purulent thrombosis, and Dialister platelets were isolated from the three blood culture bottles by pure culture (68). Dialister platelets may have a certain correlation with thrombosis, which also reflects the reliability of the results of this study.

We also found that Lactobacillales was genetically correlated with DVT. Lactobacillales are a functional group of acid-resistant and gram-positive bacteria that are mainly divided into bacilli and the phylum Firmicutes, which play an important role in human nutrition and exist as symbionts in the gut (69). Lactic acid bacteria rely on toll-like receptor 2 and protein kinase C, which have protective effects on intestinal epithelial barrier function (70). During HIV infection, maintaining the ratio of gut Lactobacillales is beneficial for restoring and protecting the immune system (71). Stool Lactobacillus counts in type 2 diabetes patients are low (72), and Enterococcus hirae WEHI01 can improve the symptoms of type 2 diabetes by increasing the abundance of Lactobacillales in rats (73). Lactobacillus casei can improve the symptoms of experimental RA by suppressing the inflammatory immune response and can be used as an effective nutritional regulator to treat OA by reducing pain, inflammatory responses, and articular cartilage degradation (74). Although there have been previous case reports of PVT and liver abscesses caused by Lactococcus lactis (75), there is no evidence of an association between Lactobacillales and DVT. Our findings suggest that the abundance of Lactobacillales has a suggestive genetic correlation with DVT, providing a new direction for further research on DVT.

Studies have found that traditional Chinese medicines (TCMs) can differentially modulate gut microbiota based on their nature. Antidiarrheal TCMs of different natures showed distinct effects on the gut microbiota. Hot-natured TCMs have no influence on the gut microbiota, warm-natured TCMs have a moderate influence, cool-natured TCMs have a strong influence, and cold-natured TCMs substantially change the structure of the gut microbial community (76). Dietary nutrients have regulatory effects on the gut microbiota. Current research shows that dietary fibers, including arabinoxylans, galacto-oligosaccharides, inulin, and oligofructose, promote a range of beneficial bacteria and suppress potentially detrimental bacterial species (77). Modulation of gut microbes will be increasingly used to promote overall health and help treat diseases (78). The gut microbiome is an important consideration in cardiovascular health and disease, with gut barrier defects leading to the transfer of gut microbes to the aorta to trigger inflammation and microbe-derived metabolites that induce inflammatory signaling pathways and renal dysfunction (79). Moreover, (poly)phenols have the capacity to promote beneficial gut bacteria through direct and collaborative bacterial utilization and their inhibitory action on potentially pathogenic species. The (poly)phenol duplibiotic effect could participate in blunting metabolic disturbance and gut dysbiosis and has therapeutic potential (80). Dietary cellulose can prevent gut inflammation by modulating lipid metabolism and the gut microbiota (81). In addition, studies have found that gut microbes can modulate platelet function and thrombosis risk (82). Gut microbes directly regulate platelet hyperreactivity and thrombotic potential through the production of trimethylamine N-oxide (TMAO). Studies in animal models have found that increasing gut microbes and dietary nutrients produced by TMAO can modulate platelet hyper-responsiveness and thrombotic potential *in vivo*. Trimethylamine (TMA) is a precursor of TMAO production in the liver and is abundant in animal products such as eggs, liver, beef, and pork. The dietary supplement choline is a nutrient that contains TMA. In mice, dietary supplementation with TMAO or choline increased plasma TMAO levels, ADP-induced platelet aggregation and shortened the rate of clot formation *in vivo*. Unrecognized mechanistic links between specific dietary nutrients, gut microbes, platelet function, and thrombotic risk provide new potential therapeutic targets and nutritional interventions for the prevention of cardiovascular events and DVT (82). TMAO levels could have clinical utility for identifying individuals who might benefit from antiplatelet prophylaxis therapies, and it is speculated that targeting this microbial pathway has the potential to reduce blood clot formation without the bleeding complications of other antiplatelet therapies (82). We speculate that gut microbiota could be regulated by TCMs, dietary cellulose, and TMAO, thereby preventing the occurrence of DVT to a certain extent. However, further research is needed to confirm this hypothesis.

Our study analyzed genotype data from GWAS summary datasets with a large sample size. LDSC has a strong genetic estimation ability, and MR has a strong genetic causal inference ability, so the results of our study are reliable. However, this study has certain limitations. First, all subjects in this study were of European descent, and the results of this study should be interpreted with caution when extended to individuals of different ethnicities. Second, the SNP set related to the gut microbiota comes from previously published GWAS research, and the GWAS data on the gut microbiota available on different platforms are very limited; therefore, we could only analyze limited gut microbiota at certain taxonomic levels. As some gut microbiota may have been overlooked, more GWASs of the gut microbiota are needed to illustrate the interaction between gut microbiota and host genetics. Third, as the data are from different populations, the results may be affected by some confounding factors, and further research on data from the same population may be needed in the future.

## Conclusion

Based on the GWAS summary data from a large population, we analyzed the genetic correlation between DVT and the gut microbiota and found that Streptococcaceae, Dialister, Streptococcus, and Lactobacillales have suggestive genetic correlations with DVT. There was a positive genetic causal relationship between Streptococcaceae and DVT. Our results provide a new direction for further research on DVT. We will continue to study the specific relationship between these gut microbes and DVT and explore their potential value in the pathogenesis and prevention of DVT.

## Data availability statement

Publicly available datasets were analyzed in this study. This data can be found here: https://broad-ukb-sumstats-us-east-1.s3.amazonaws.com/round2/additive-tsvs/20002_1094.gwas.imputed_v3.both_sexes.tsv.bgz and here: https://doi.org/10.5523/bris.22bqn399f9i432q56gt3wfhzlc.

## Author contributions

PX and MY designed the study. MY, PL, and FZ analyzed the data and interpreted the results. KX and RF provide the software support. MY wrote and edited the manuscript. PX provided the foundation support. All authors read and approved the final manuscript.

## Footnotes

1
https://github.com/bulik/ldsc

2
http://www.phenoscanner.medschl.cam.ac.uk/phenoscanner

3
https://ldlink.nci.nih.gov/
